# Further validation that claims data are a useful tool for epidemiologic research on hypertension

**DOI:** 10.1186/1471-2458-13-51

**Published:** 2013-01-18

**Authors:** Baylah Tessier-Sherman, Deron Galusha, Oyebode A Taiwo, Linda Cantley, Martin D Slade, Sharon R Kirsche, Mark R Cullen

**Affiliations:** 1 Yale Occupational and Environmental Medicine Program, Yale University School of Medicine, New Haven, CT, USA; 2 Department of Internal Medicine, Stanford University School of Medicine, Stanford, CA, USA

**Keywords:** Hypertension, Administrative data, Validation, Specificity and sensitivity, Medical records

## Abstract

**Background:**

The practice of using medical service claims in epidemiologic research on hypertension is becoming increasingly common, and several published studies have attempted to validate the diagnostic data contained therein. However, very few of those studies have had the benefit of using actual measured blood pressure as the gold standard. The goal of this study is to assess the validity of claims data in identifying hypertension cases and thereby clarify the benefits and limitations of using those data in studies of chronic disease etiology.

**Methods:**

Disease status was assigned to 19,150 employees at a U.S. manufacturing company where regular physical examinations are performed. We compared the presence of hypertension in the occupational medical charts against diagnoses obtained from administrative claims data.

**Results:**

After adjusting for potential confounders, those with measured blood pressure indicating stage 1 hypertension were 3.69 times more likely to have a claim than normotensives (95% CI: 3.12, 4.38) and those indicating stage 2 hypertension were 7.70 times more likely to have a claim than normotensives (95% CI: 6.36, 9.35). Comparing measured blood pressure values identified in the medical charts to the algorithms for diagnosis of hypertension from the claims data yielded sensitivity values of 43-61% and specificity values of 86–94%.

**Conclusions:**

The medical service claims data were found to be highly specific, while sensitivity values varied by claims algorithm suggesting the possibility of under-ascertainment. Our analysis further demonstrates that such under-ascertainment is strongly skewed toward those cases that would be considered clinically borderline or mild.

## Background

Healthcare claims data play a vital role in research aimed at crafting health policy, establishing medical standards of care, and understanding disease etiology. While the ideal methods of collecting clinical data for epidemiologic research are medical chart review and physical examination [1], these more direct approaches are laborious and expensive, and thus the use of administrative datasets is becoming increasingly common. Most often derived in the United States from Medicare, Medicaid, and Veterans Administrative populations, these datasets are used extensively in health services research and pharmacoepidemiology, but are increasingly of interest for etiologic studies [2].

Several studies have evaluated the use of claims data in accurately identifying cases of hypertension [1,3-9], though very few have used serial blood pressure measurements abstracted from chart data as the source of comparison. Instead, research has focused on using self-reported hypertension as a reference standard for assessing the accuracy of claims data and chart review data [5,6,9-11]. However, the use of self-report data presents significant limitations in any estimates of the sensitivity and specificity of other data sources. Lack of medical knowledge, medical terminology as well the tendency for those with hypertension to underreport all contribute to a significant skepticism about self-reported data as a validating data source [9,12]. Additional issues arising in the literature include: the impact of differential health and drug benefit packages within study population [9]; limitations of physician notes for diagnostic purposes [13]; and exclusive attention to cases in evaluating the accuracy of patient claims [4].

With the vast majority of healthcare dollars being spent on chronic conditions, it is essential for researchers to continue to assess the benefits and limitations of administrative datasets for the scientific study of the causes and outcomes of chronic diseases, as well as to better understand which strategies of disease ascertainment are the most useful in conducting etiologic research. The objective of this study is to assess the use of administrative data for identifying cases of hypertension in a large industrial cohort which has been under long-term scrutiny. Using medical claims data, we examine the presence of hypertensive disease in employees with measured blood pressure readings from routine physical examinations at the workplace as a job safety requirement.

## Methods

The study company is a multinational producer of aluminum and related products, operating in 24 states and around the world. The US workforce has varied from over 50,000 to around 30,000, with the majority of workers in hourly manufacturing jobs. Notably, although workers may select from a menu of health benefits in terms of cost, there is only a single preferred provider organization (PPO) network available at each location; managed care organization (MCO) alternatives are offered at a small handful of locations. Because the offered plans are rich in terms of coverage, the vast majority of employees and their families enroll in them.

### Data sources

All data are available as part of a unique academic-corporate partnership that began in 1997 for the purpose of developing and implementing workplace safety and occupational health policies for this large, multi-site aluminum manufacturer. The research agreement allows the investigators regularly updated company databases which are then de-identified and linked. The databases have been described in greater detail in previous publications [2,14]. Briefly, they include the following:

#### Human resources

Provided annually, this database contains all employee demographic information, including date of birth, race, and sex. Files are created at the start of employment and document all changes in job title, job grade, job status (active, on leave, retired), job category (hourly or salary), and plant location.

#### Occupational health screening

This database provides basic health screening information for employees who participate in fitness-for-duty evaluations at the start of employment and medical surveillance programs which are typically performed every three years of employment. Although the extent of screening an employee participates in varies by job, all employees in this study participated in at least one medical screening program in which blood pressure measurements were collected. Occupational health data were provided to the investigators in one of two ways: some plants maintain an electronic database, to which the investigators have access, of data gathered from mandatory health screenings, while other plants merely record such data in the employees' paper charts. Beginning in 2002 the investigators, in an attempt to collect more comprehensive data on employee health risk factors, began abstracting health data from the individual plant medical departments. Data collected include smoking history, cholesterol, blood pressure, height, weight, education status, and marital status. Chart notes, such as physician comments regarding diagnosis or history of hypertension, are not available.

#### Insurance claims database

Investigators annually receive medical and pharmacy claims from a central data processing center, which in turn receives the data from each individual third party administrator. Data include ICD-9 codes for disease diagnosis [15] and National Drug Codes (NDC) for prescription information [16]. Data on date of service, provider type, and provider location are also available. These data are complete for almost all employees enrolled in the PPO plan.

#### Data linkage

Databases were linked by using an encrypted unique identifier created by the investigators to ensure human subject privacy.

### Study sample

A study set was created by first selecting all active employees who had one or more systolic (SBP) and diastolic (DBP) blood pressure measurements in their occupational health screening records for the period 2003 – 2009 and were enrolled in a PPO health insurance plan. Plants represented in this sample encompassed all aspects of the company’s sectors, including smelting and fabricated aluminum products, and employees were included regardless of job.

We examined the cohort in two ways: first, actual blood pressure measurements from the occupational health records were categorized into four groups: normotensive (SBP <120 and DBP <80), pre-hypertensive (SBP of 120–139 or DBP of 80–89), stage 1 hypertensive (SBP of 140–159 or a DBP of 90–99), or stage 2 hypertensive (SBP of ≥160 or a DBP ≥100). If an employee’s blood pressure values overlapped between 2 groups, he was placed in the higher group. Second, each employee was classified into one of two categories: normal blood pressure or high blood pressure based on the pattern of their measurements in their medical charts. If an employee had at least one high blood pressure reading, they were classified as having high blood pressure; if they only had normal readings, they were classified as having normal blood pressure. The borderline category was omitted from this part of the analysis to allow us to calculate sensitivity and specificity values.

Administrative claims data (defined as a physician billing and/or hospital admission of hypertension as the primary diagnostic code, 401.XX-405.XX) for the study individuals for the same time period were then examined by defining hypertension using the following possible algorithms: 1) at least one medical claim based on a face-to-face examination or hospitalization or 2) at least two separate medical claims. No time gap was required between separate medical claims.

### Statistical analysis

A Monte Carlo logistic regression was performed on all blood pressure measurements to elucidate the predictors of having at least one claim for hypertension while adjusting for covariates. The simulation was chosen whereby a single, randomly selected blood pressure reading was included for ordinal logistic regression analysis. This approach was conducted in order to account for the varied number of repeated blood pressure measurements per employee. Further, it allowed us to tease out the potentially confounding influence of those in the borderline category, as well as control for disease severity and other individual covariates. The resulting adjusted odds ratio estimate for the effect of blood pressure category on having a hypertensive claim was recorded. This simulation was conducted 10,000 times in order to determine the central tendency for the odds ratio as well as the 95% confidence interval.

Frequency (2×2) tables were then constructed for each algorithm used to define employees’ hypertension status from the administrative claims data. Sensitivity was defined as the proportion of employees with high blood pressure measurements in their occupational health records that were identified as hypertensive by a claims-based algorithm. Specificity was calculated as the proportion of employees with normal blood pressure measurements in their medical charts and not identified by a claims-based algorithm as having hypertension. The positive predictive value (PPV) of using claims was calculated as the proportion of employees with claims for hypertension who had normal blood pressure measurements in their medical charts. The negative predictive value (NPV) of using claims was calculated as the proportion of employees without a claim for hypertension who had normal blood pressure measurements in their medical charts.

Statistical analyses were performed with SAS version 9.2.

## Results

**Figure 1:**
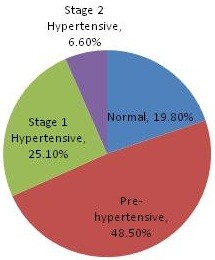
Classification of Blood Pressure Measurements (n = 49,628).

Our final cohort consisted of 19,150 employees, representing over 30 U.S. plants for the period 2003–2009. Table 1 shows the demographics for the cohort. The mean age for the cohort was 43.8 years, with 85.3% male and 80.8% Caucasian. 73.7% of the cohort consisted of hourly employees with an average company tenure of close to 14 years. The mean number of clinic encounters per employee where a blood pressure measurement was recorded was 2.6, with 30% of employees having more than 3 encounters within the study period. Figure 1 shows the breakdown of individual blood pressure measurements by disease classification. Out of the 49,628 measurements, nearly half were pre-hypertensive readings, 20% were in the normal range, 25% indicated stage 1 hypertension, and 7% stage 2 hypertension. Less than 1% of all the medical claims for hypertension were in the form of hospital admissions, the vast majority being physician visits.

**Table 1:** **Demographics of study cohort**, **n** = **19**,**150**

	
Age*, mean(sd)	43.8(10.6)
Sex	
Female	2812(14.7%)
Male	16335(85.3%)
Unknown	3(0.0%)
Ethnicity	
African-American	2152(11.2%)
American Indian	156(0.8%)
Asian/Pacific Islander	225(1.2%)
Caucasian	15464(80.8%)
Hispanic	1125(5.9%)
Unknown	28(0.1%)
Employee Type	
Hourly	14113(73.7%)
Salary	5034(26.3%)
Unknown	3(0.0%)
Tenure* (yrs), mean(sd)	13.6(12.2)
Average No. of Chart Measurements, mean(sd)	2.6(4.2)
Frequency of Chart Measurements per Employee	
1	8455(44.2%)
2	4680(24.4%)
3	2529(13.2%)
4	1321(6.9%)
5+	2165(11.3%)

*Age and tenure were calculated using the date of the employee's first blood pressure measurement.

Table 2 shows the results of the Monte Carlo logistic regression model. All employees and all blood pressure readings were included in this analysis. After controlling for age, sex, race, and employee type, there were significantly increased odds of having a medical claim for hypertension as disease severity increases. Those with blood pressure values indicating stage 1 hypertension were 3.69 times more likely to have a claim for hypertension compared to those with normal blood pressure values (95% CI: 3.12, 4.38), and those with blood pressure values indicating stage 2 hypertension were 7.70 times more likely to have a claim for hypertension compared to those with normal blood pressure values (95% CI: 6.36, 9.35).

**Table 2:** Predictors of having a claim for hypertension, Monte Carlo logistic model, n = 19,150

**Parameter**	**Level**	**Odds ratio**				
		**Estimate**	**L95%**	**U95%**		
Blood pressure	Normal	1.00	Reference				
	Borderline	1.92	1.63	2.26			
	Stage 1	3.69	3.12	4.38			
	Stage 2	7.70	6.36	9.35			
Employee Type	Salaried	0.74	0.74	0.75			
	Hourly	1.00	Reference				
Age	per year	1.08	1.08	1.08			
Race	Non-white	1.38	1.36	1.39			
	White	1.00	Reference				
Sex	Female	0.85	0.84	0.86			
	Male	1.00	Reference				

Only employees with high or normal blood pressure measurements were included for the sensitivity and specificity analyses. 54% (or 10,415 employees) met this criterion, while 46% (8,735 ‘borderline’ employees) were excluded. 40.6% had high blood pressure measurements in their charts, while 13.8% were in the normal range and 45.6% did not fit either category and were labeled as borderline. Of those borderlines that were excluded, 72% had only pre-hypertensive readings (defined as SBP between 120–139 or DBP between 80–89), while the remaining 28% had mixed normal/pre-hypertensive readings. Males were more likely to have hypertensive readings in their charts compared with females (p < 0.0001), and hourly employees were more likely to have hypertensive readings in their charts compared with salaried employees (p < 0.0001). Those in the high blood pressure category had an average tenure of 15 years, whereas those in the normal category had an average tenure of 11 years. Likewise, the average age was 5 years older in the hypertensive category.

As Table 3 shows, the claims data captures between 43–52% of those employees who have high blood pressure readings in their occupational health records. Using the looser algorithm of ≥1 medical claim, we calculate a sensitivity of 52%, while using the more stringent algorithm of ≥2 medical claims results in a sensitivity of 43%. Specificity ranged from 86% for employees with ≥1 medical claim to 90% for employees with ≥2 medical claims. Table 3 also shows the results of the PPV and NPV calculations. The PPV ranged from 92–93% and NPV ranged from 35–38%.

**Table 3:** Association between measured blood pressure readings and claims data

**Claims algorithm**	**N**	**Sensitivity**	**Specificity**	**Pos.predictive value**	**Neg.predictive value**
≥ 1 medical claim	4026	51.77%	85.87%	91.52%	37.67%
≥ 2 medical claim	3339	42.94%	90.41%	92.96%	34.97%

In an attempt to avoid inaccurate classification of cases due to blood pressure variability [17], we conducted a subanalysis of our cohort using a more restrictive case definition. Only employees who had two or more consistent blood pressure readings during this period, i.e. their blood pressure measurements were persistently normal or hypertensive, were included (n = 1766). For example, if employees had three readings and only the 2nd and 3rd readings were categorized as high, that employee was not included in the cohort. Similar to the Monte Carlo, no time gap was required between measurements. Table 4 shows these results. Sensitivity for the one and two claims algorithms were 61% and 52% and specificity values were 94% and 89%, respectively. Although the PPV values were comparable to those of the larger cohort, the NPV improved significantly, ranging from 56%–60%.

**Table 4:** Association between measured blood pressure readings and claims data for subanalysis cohort

**Claims algorithm**	**N**	**Sensitivity**	**Specificity**	**Pos.predictive value**	**Neg.predictive value**
≥ 1 medical claim	654	61.29%	88.70%	89.22%	60.02%
≥ 2 medical claim	555	52.01%	93.56%	92.50%	56.09%

## Discussion

In this study, we examined the relationship between measured blood pressure values and administrative medical claims for hypertension. By first including all measurements for all employees in a multivariate simulation, we found the odds of having a claim of hypertension were significantly increased for those employees with actual blood pressure reading suggesting stage 1 hypertension as compared to those with normal measurements, and increased even more dramatically for those with readings suggesting stage 2 hypertension. This model allowed us to control for several potentially confounding variables such as an unequal number of repeated measures per employee, age, sex, employee type and race.

We next assessed the accuracy of two algorithms for defining hypertension using claims data compared to actual blood pressure measurements from occupational health records. Our results show a sensitivity of 42–52% and a specificity of 86–90% depending on the algorithm used. These results are comparable to other studies utilizing different methods including self-reported survey data on hypertension and review of medical records to validate claims data. These studies have reported sensitivity values ranging from 52–75% and specificity values ranging from 75–94% [1,4,7-10,13].

In a study of claims-based algorithms for identifying chronic medical conditions, Rector et al. reported a sensitivity and specificity of 52% and 91% respectively in diagnosing hypertension using a combination of one medical claim and one pharmacy claim when compared with self-reported surveys [9]. Tu et al. also reported sensitivity of 73% and specificity of 95% utilizing two outpatient claims for detecting hypertension compared with physician-assigned diagnosis [10]. The sensitivity and specificity dropped to 64% and 94% respectively, when the same combination (2 outpatient claims for detecting hypertension) was compared to self-reported survey data.

In our claims dataset, the more sensitive algorithm was to use at least one medical claim for identifying disease; changing the algorithm to at least two medical claims decreased the sensitivity and only modestly increased the specificity. We initially conducted this analysis using all employees who had at least one high blood pressure measurement as the source of comparison; however, we repeated the analysis using a more stringent case definition of classifying employees who had two or more consistent readings during this period. The study population dropped from 10,415 in the initial analysis to 1766 in the subanalysis. Although the specificity values did not significantly change, sensitivity values improved.

All analyses were conducted regardless of whether or not the employee was using prescription medication for lowering blood pressure, an obvious reason for a false positive. Because these medications are commonly used for conditions not associated with high blood pressure, removing those employees from the analysis would have limited impact. Indeed in a sensitivity analysis where we eliminated normotensives on blood pressure lowering medications, our results did not change substantively for the ≥1 and ≥2 medical claim algorithms: sensitivity values remained unchanged, specificity values increased to 95.51% and 98.44%, positive predictive values increased to 97.55% and 98.96%, and negative predictive values decreased modestly to 36.41% and 33.38%, respectively. With nearly 70% of false positives using blood pressure lowering medications, the specificity rates reflected in this study are likely lower than previously found because of this phenomenon.

This study has certain limitations. First, although we are confident from our multivariate model that sex, race and employee type are unlikely to be the source of significant confounding, we are less confident about job type, as only certain jobs require regular examinations which in turn might lead to referral for treatment, while other jobs have no such “screening”. Second, by choosing a cross-sectional study design, we were able to take advantage of a large and robust dataset that includes a varied number (sometimes only one) of intermittent blood pressure readings per person. However, by allowing for the inclusion of all individuals with at least one blood pressure reading, we sacrificed our ability to examine the data in a temporal manner. Specifically, those who entered the cohort later in the study period have shorter windows of opportunity for having a claim in the administrative dataset. This likely contributed to conservative estimates. The third limitation was the unbalanced nature of the data. In particular, not all employees had the same number of blood pressure measurements. The Monte Carlo simulation mitigated the problem of increased probability of a hypertensive reading with increased measurements. Lastly, inherent in the use of claims data is the potential for missing claims due to physician preference in choosing a diagnostic code for patients with comorbidities. In this study, the frequency of hypertension claims suggests that such a bias is not likely leading to an underestimation of disease.

## Conclusions

This study not only provides evidence of the potential value of using administrative claims in epidemiologic research on hypertension, but also demonstrates that the inevitable under-ascertainment from using claims data to study hypertension is not at random. Those most likely to be missed through the use of claims driven analyses are those with the least severe disease. Conversely, specificity appears to be lost in large measure because of treatment. One of the major strengths of this study was the use of actual blood pressure measurements from employee health records as the comparison data source. This is likely superior to self-reported survey data for verification of hypertension and is more comparable to using physician diagnosis of hypertension abstracted from medical records as the gold standard.

While the wide availability of administrative health datasets provides researchers with boundless opportunities for analyzing data, analyzing subsets of claims data, as was done in this study, should allow investigators to more thoroughly understand the strengths and limitations of using claims datasets for etiologic research in hypertension. Although our high specificity values demonstrates the value of using claims data in defining a cohort of hypertensives, using the claims data to identify cases of hypertension as a covariate in statistical models may introduce under-ascertainment. Mitigating this, however, our analysis strongly suggests that such under-ascertainment is skewed toward those cases that would be considered clinically borderline or mild.

## Competing interests

There are no competing interests to declare with this submission, and this manuscript has been read and approved by all authors. The authors acknowledge that this research was supported by a grant from the National Institute on Aging and a contract with Alcoa, Inc. Funding agencies had no direct influence on the design, analysis, or preparation of this manuscript for publication. Ethical approval for this study was granted by the Yale Human Investigations Committee.

## Authors’ contributions

In the preparation of this manuscript, OAT, MRC, and BTS conceived of and developed the study design. BTS, DG, and MDS conducted the data analysis, and BTS, LC, and MRC participated in data interpretation and drafting the final paper. SRK was involved in project coordination, data interpretation, and critical revisions. All authors have read and approved the final manuscript.

## NIA data sharing

As an alternative to providing a de-identified data set to the public domain, we allow access for the purpose of re-analyses or appropriate “follow-on” analyses by any qualified investigator willing to sign a contractual covenant with the host Institution limiting use of data to a specific agreed upon purpose and observing the same restrictions as are limited in our contract with Alcoa, such as a 60-day manuscript review for compliance purposes.

## Pre-publication history

The pre-publication history for this paper can be accessed here:

http://www.biomedcentral.com/1471-2458/13/51/prepub

## References

[B1] Quan H Khan N Hemmelgarn BR Tu K Chen G Campbell N Hill MD Ghali WA McAlister FA Validation of a Case Definition to Define Hypertension Using Administrative Data Hypertension 2009 54 6 1423 1428 http://hyper.ahajournals.org/content/1454/1426/1423 10.1161/HYPERTENSIONAHA.109.139279 19858407

[B2] Cullen MR Vegso S Cantley L Galusha D Rabinowitz P Taiwo O Fiellin M Wennberg D Iennaco J Slade MD Use of medical insurance claims data for occupational health research J Occup Environ Med 2006 48 10 1054 1061 10.1097/01.jom.0000241049.23093.a4 17033505

[B3] Lix L Yogendran M Burchill C Metge C McKeen N Moore D Bond R Defining and validating chronic diseases: An administrative data approach 2006 Winnipeg: Manitoba Centre for Health Policy

[B4] Quam L Ellis LB Venus P Clouse J Taylor CG Leatherman S Using claims data for epidemiologic research. The concordance of claims-based criteria with the medical record and patient survey for identifying a hypertensive population Medical Care 1993 31 6 498 507 http://www.ncbi.nlm.nih.gov/pubmed/8501997 10.1097/00005650-199306000-00003 8501997

[B5] Robinson JR Young TK Roos LL Gelskey DE Estimating the burden of disease. Comparing administrative data and self-reports Medical Care 1997 35 9 932 947 http://www.ncbi.nlm.nih.gov/pubmed/9298082 10.1097/00005650-199709000-00006 9298082

[B6] Muhajarine N Mustard C Roos LL Young TK Gelskey DE Comparison of survey and physician claims data for detecting hypertension J Clin Epidemiol 1997 50 6 711 718 http://www.sciencedirect.com/science/article/B716T784-713RJGH727-C/712/711be0517e0513a2537cdac0511acd0062a0516c0510af0513 10.1016/S0895-4356(97)00019-X 9250269

[B7] Borzecki AM Wong AT Hickey EC Ash AS Berlowitz DR Identifying hypertension-related comorbidities from administrative data: what's the optimal approach? Am J Med Qual 2004 19 5 201 206 10.1177/106286060401900504 15532912

[B8] Wilchesky M Tamblyn RM Huang A Validation of diagnostic codes within medical services claims J Clin Epidemiol 2004 57 2 131 141 10.1016/S0895-4356(03)00246-4 15125622

[B9] Rector TS Wickstrom SL Shah M Thomas Greeenlee N Rheault P Rogowski J Freedman V Adams J Escarce JJ Specificity and Sensitivity of Claims-Based Algorithms for Identifying Members of Medicare + Choice Health Plans That Have Chronic Medical Conditions Health Serv Res 2004 39 6p1 1839 1858 http://onlinelibrary.wiley.com/doi/1810.1111/j.1475-6773.2004.00321.x/abstract 10.1111/j.1475-6773.2004.00321.x 15533190 PMC1361101

[B10] Tu K Campbell NRC Chen Z-L Cauch-Dudek KJ McAlister FA Accuracy of administrative databases in identifying patients with hypertension 2007 1 1 e18 e26 PMC2801913 20101286

[B11] Hebert PL Geiss LS Tierney EF Engelgau MM Yawn BP McBean AM Identifying Persons with Diabetes Using Medicare Claims Data Am J Med Qual 1999 14 6 270 277 http://ajm.sagepub.com/content/214/276/270.abstract 10.1177/106286069901400607 10624032

[B12] Goldman N Lin IF Weinstein M Lin Y-H Evaluating the quality of self-reports of hypertension and diabetes J Clin Epidemiol 2003 56 2 148 154 http://www.sciencedirect.com/science/article/B146T184-485D140HC-147/142/100adfe154f436e295c10747cd10740accbbbb 10.1016/S0895-4356(02)00580-2 12654409

[B13] Bullano MF Kamat S Willey VJ Barlas S Watson DJ Brenneman SK Agreement Between Administrative Claims and the Medical Record in Identifying Patients With a Diagnosis of Hypertension Medical Care 2006 44 5 486 490 http://journals.lww.com/lww-medicalcare/Abstract/2006/05000/Agreement_Between_Administrative_Claims_and_the.05014.aspx 10.1097/01.mlr.0000207482.02503.55 16641668

[B14] Pollack KM Agnew J Slade MD Cantley L Taiwo O Vegso S Sircar K Cullen MR Use of employer administrative databases to identify systematic causes of injury in aluminum manufacturing Am J Ind Med 2007 50 9 676 686 http://onlinelibrary.wiley.com/doi/610.1002/ajim.20493/abstract 10.1002/ajim.20493 17676586

[B15] National Center for Health Statistics International Classification of Diseases, Ninth Revision, Clinical Modification 1988 Washington, DC: Public Health Service, U.S. Dept of Health and Human Services

[B16] National Drug Code Directory http://www.accessdata.fda.gov/scripts/cder/ndc/default.cfm

[B17] Turner MJ van Schalkwyk JM Blood pressure variability causes spurious identification of hypertension in clinical studies: a computer simulation study Am J Hypertens 2008 21 1 85 91 http://www.ncbi.nlm.nih.gov/pubmed/18091749 10.1038/ajh.2007.25 18091749

